# Stochastic driver compensation for road gradients across vehicle classes: a machine learning approach

**DOI:** 10.1038/s41598-026-51143-4

**Published:** 2026-05-28

**Authors:** Kai Yuan, Hongye Xing, Weihua Zhang, Rui Jiang, Shixiong Jiang

**Affiliations:** 1 School of Automotive and Transportation Engineering, Hefei University of Technology, Hefei, 230009 Anhui China; 2 School of Civil Engineering, Hefei University of Technology, Hefei, 230009 Anhui China; 3 School of Systems Science, Beijing Jiaotong University, Beijing, 100044 China; 4 College of Urban Transportation and Logistics, Shenzhen Technology University, Shenzhen, 518118 China

**Keywords:** Slope, Compensation acceleration, Intra-driver variations, Expectation-maximization algorithm, Engineering, Mathematics and computing

## Abstract

Slopes often emerge as traffic bottlenecks, yet not all slopes lead to congestion. The relationship between slope capacity and factors like grade and length is complex and non-linear. Accurately estimating road slope capacity and mitigating traffic congestion remain challenges in traffic management. Drivers instinctively adjust vehicle acceleration or braking to counteract gravity, influencing vehicle speed and road capacity. However, traditional models often overlook these compensatory adjustments, leading to inaccurate predictions. This study introduces a novel approach using vehicle trajectory data and the Expectation-Maximization (EM) algorithm to estimate driver compensations on slopes. The algorithm separates observed acceleration into baseline (flat road) and compensatory components. Data from field experiments with cars, trucks, and buses reveal that compensatory acceleration decreases with speed and remains predictable across different slopes. These findings enhance our understanding of slope impacts on traffic flow and provide valuable insights for traffic management and infrastructure design.

## Introduction

**Fig. 1:**
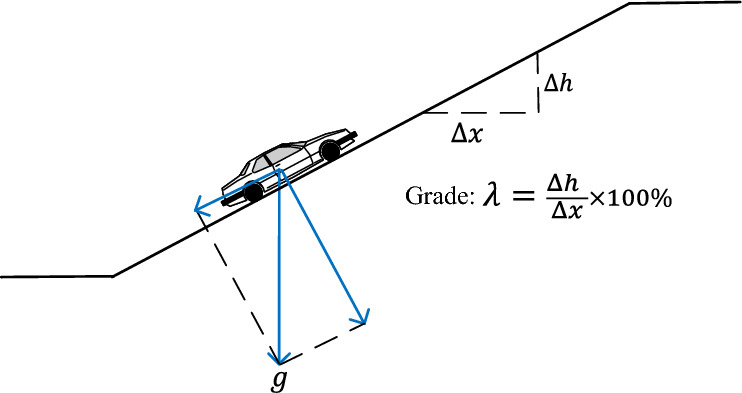
Gravitational acceleration *g ≈* 9.8m/$\hbox {s}^{2}$ would generate a resistance force that reduces the vehicle acceleration on a slope with grade *λ*.

Longitudinal slope describes an angle at which a freeway vertical curve inclines or declines. The term “grade” is used to describe the longitudinal slope with a unit %, see Fig. 1. The longitudinal slope indicates an uphill or downhill road section. In Japan, nearly 60% of traffic congestion occurs at uphills or the uphill section at sags^1,2^. In addition, the acceleration profile on slopes would result in extra fuel consumption and gas emissions^3–5^. According to China’s Technical Standard of Highway Engineering (JTG B01-2014)^6^, the maximum longitudinal gradient for expressways ranges from 3% to 5% depending on the design speed: 3% for a design speed of 120 km/h, 4% for 100 km/h, and 5% for 80 km/h or below.

### Literature review: compensation behaviors on slopes

Many empirical studies have shown that slopes can be typical bottlenecks and their capacity may be lower than that of horizontal sections^7,8^. A resistance force due to the gravity would tend to reduce the acceleration of vehicles on a uphill slope, see Fig. 1. Consequently, drivers would adjust the throttle or brake pedal to counteract gravitational effects, which is the compensation behavior. As found in empirical studies, drivers seems cannot always fully compensate for the gravitational effects on slopes by pressing throttle^9–13^. Prior research^12,13^ indicates car drivers typically offset about half of gravitational effects on slopes, while truck drivers only compensate for approximately 5% of the loss. That finally results in macroscopic traffic flow phenomena on uphill road sections, e.g., the reduction of capacity^9,14–16^ and the exacerbation of oscillation growth^17^.

**Fig. 2:**
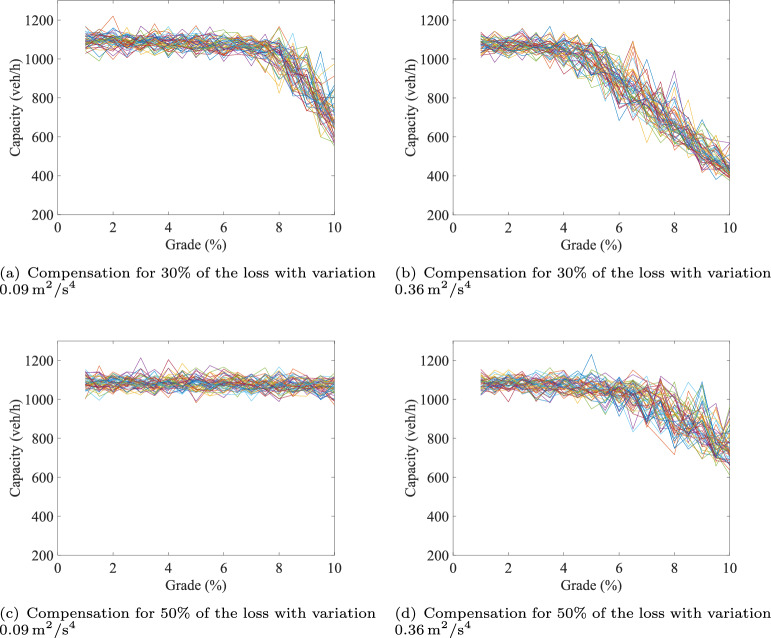
Non-linear relation between the road grade and the capacity. The capacity would reduce only when the grade exceeds a threshold. In (c), the slope is not a bottleneck since the capacity does not decrease over all grades. Results are generated by simulations. The simulation scenario is a slope with length 400m. Stochastic nature of compensation accelerations are considered. The compensation acceleration is generated using normal distribution. The expected value and variance are constant in every simulation scenario.

Present research on the behavioral mechanisms behind traffic flow on uphill road sections is inadequate. The results in Fig. 2 rely on simplified assumptions about compensation acceleration in numerical simulations. Though the proposed non-linear relationship is plausible, it is still unclear whether road grade significantly reduces capacity across all grades and slope lengths, or if there is a specific threshold (e.g., road grade or length) beyond which capacity reduction becomes considerable. Addressing these questions is crucial for understanding the impact of slopes on traffic flow and capacity. Such understanding is vital for traffic planning and management, enabling more precise predictions of traffic conditions on sloped roads. It also benefits road infrastructure design and improvement by helping to address potential bottlenecks effectively. However, to the best of the authors’ knowledge, these questions remain unanswered due to the lack of a connection between traffic flow phenomena on slopes and driver compensation behavior.

Some researchers have argued that identifying drivers’ speed intentions, such as acceleration and deceleration, is important for understanding driver behavior. However, existing studies have mainly focused on recognizing typical speed intentions. They have not fully considered how various factors in the driving environment (e.g. road gradient) affect recognition of these intentions^18^. Some researchers suggest that when vehicles ascend slopes, drivers need a specific time delay to adjust the throttle position to fully counteract the increased resistance force^10^. Goñi Ros et al.^15^ incorporate this time-delay mechanism into a car-following model at sags, assuming compensation acceleration rises linearly over time. Their model can replicate some features of congestion formation on slopes, like congestion forming at the slope’s end, implying that compensatory acceleration may vary over time and space. Yet, empirical data validation is still missing. The studies in Xu and Laval^12^ and Tu and Laval^13^ is based on empirical data, but they overlook the stochastic nature of compensation behavior.

Compensation behavior, much like other human behaviors, is stochastic in nature or is marked by some level of uncertainty. When we delve into the stochastic behavioral mechanisms underpinning traffic phenomena such as capacity and instability, two mechanisms need to be explored: inter-driver spread^19^ and intra-driver variation^20^. Inter-driver spread speaks to the heterogeneity among different vehicle-driver units, whereas intra-driver variation defines the stochastic characteristics of condition-dependent driver behaviors within the same vehicle-driver unit^21^. The analysis of vehicle-driver types from different perspectives is important for improving traffic micro-simulation and for gaining a better understanding of the complex mechanisms behind traffic phenomena^22,23^. The heterogeneity of vehicle-driver types is relevant to the study of capacity on slopes. For example, grades can considerably impact trucks and buses because of their limited acceleration abilities. On slopes, different drivers would conduct different compensation behavior, which consequently impact road capacity. However, to investigate the inter-driver spread of compensation behaviors, we must first quantify the intra-driver variation of this behavior. By comparing the intra-driver variation of different drivers, we can then delve into the inter-driver spread. Hence, this work aims to develop a method to quantify the intra-driver variation of compensation behaviors from empirical data.

### Research objectives and contributions

A key challenge lies in collecting compensatory acceleration data on upgrade road sections. To the best of our knowledge, no prior studies have systematically gathered such data or analyzed the interplay between compensatory driving behavior and slope properties (e.g., grade and length) using real-world data.

This paper introduces a new framework for quantifying the stochastic compensation acceleration on slopes-a variable that prior studies have not been able to measure directly. We test and validate this framework using a controlled, small-scale dataset, intended as a proof of concept. The numerical results should therefore be viewed as an illustration of the method’s application to the specific vehicles and drivers studied, rather than as a basis for generalizing about wider driving populations. The main contribution lies in the validated method, which paves the way for applying it to larger datasets in the future. Such applications could eventually yield statistically sound insights to support slope design and traffic management. Future work must prioritize data collection in this domain to develop actionable insights for slope design and traffic management.

### Outline

Towards this end, the outline of the paper is as follows: we present the compensation behavior estimation approach in Section “Methodology”. Filed experiments are conducted to collect data, see Section "Data and experiments". The compensatory behavior is estimated and analyzed in Section "Intra-driver compensation behaviors". Finally, we end this paper with conclusions and future research in Section "Conclusions and future research".

## Methodology

To fill this gap, we propose an Expectation-Maximization (EM) approach to quantify the compensation acceleration to slopes. The EM algorithm is an iterative optimization technique for estimating parameters in statistical models that incorporate hidden and observed variables^24^. This method alternates between two critical phases: the Expectation step (E-step) and the Maximization step (M-step). The method is widely used in parameter estimation problems in traffic scenarios^25–29^. This method proves especially effective when directly measuring essential variables is challenging or impossible^30,31^.

When formulating the research problem within the EM algorithm framework, it is crucial to incorporate compensation acceleration into the car-following model. To achieve this, we define the desired acceleration $a_r$, which consists of three components: (i) the desired acceleration on horizontal sections, denoted as $a_e$, (ii) the compensation acceleration, represented by $a_g$, and (iii) the deceleration due to gravity, calculated as $g\sin {\arctan \lambda }$. In Xu and Laval^12^, the compensation acceleration and the gravitational deceleration are combined as $\alpha \cdot g\sin {\arctan \lambda }$, where if *-1 < α*, the compensation acceleration is positive. In this formulation, the desired acceleration $a_r$ is the sum of $a_e$ and the integrated gravitational deceleration. While this approach helps validate the compensation behavior, it does not accurately capture the precise value of the compensation acceleration, thereby overlooking important details of the compensation dynamics. Therefore, in this work, the observed desired acceleration $a_r$ is modeled as $a_r=a_e+a_g-g\sin {\arctan \lambda }$.

In order to describe the acceleration $a_e$, we employ the Brownian Motion (BM) car-following model as proposed by Laval et al.^17^. This model demonstrates that, on average, the desired acceleration decreases linearly as the speed increases. The relation between the desired acceleration and speed has been empirically validated by Laval et al.^17^. For each specific speed, the corresponding $a_e$ follows a normal distribution. It is important to note that in the BM model, the variation in $a_e$, denoted by $\sigma _e$, remains constant across different speeds.

Yuan et al.^20^ have developed a geometric Brownian Motion (g-BM) car following model, where the variation $\sigma _e$ decreases as the speed increases. However, based on car-following experimental data, Tu and Laval^13^ have found that the acceleration error process more closely resembles Brownian motion. Therefore, in this study, we adopt a constant $\sigma _e$.

The compensation acceleration $a_g$ is also discretized by speed. For each speed interval, a log-normal distribution with parameters ($\mu _g^v, \sigma _g^v$) is assigned. The assumption of a log-normal distribution ensures that the compensation acceleration remains non-negative, which is consistent with empirical observations reported in Xu and Laval^12^.

We conduct experiments to collect vehicle trajectory data. Three types of vehicles - a private car, a light cargo van and a 39-seat coach - are tested on urban freeways, respectively. With the proposed EM-based approach, we quantify the stochastic compensation behavior for road gradients.

In this section, we first formulate the problem in Section “Problem formulation”. To ensure a clear understanding of the mathematical formulations in this work, the key symbols and notations are summarized in this section. The primary variables and parameters are listed in Table 1. A simple theoretical analysis on the impacts of compensation accelerations on traffic flow is conducted in Section "Impacts of compensation acceleration using a linear casestudy". The compensation behavior problem is formulated in the EM algorithms framework (see Section "Formulations in EM framework"), and addressed by using the EM algorithm in Section “Solution algorithms”.

### Problem formulation

This research is based on a concept of desired acceleration. The desired acceleration is defined as the acceleration drivers are willing to impose on their vehicles in the absence of a leader. The desired acceleration corresponds to the acceleration processes of a single driver on an empty road, only constrained by the engine power and the way the driver uses it. In this study, we conduct field experiments to collect the desired acceleration $a_r$, see Section "Data and experiments". On a uphill road section, the desired acceleration of a vehicle, denoted by $a_r$ on slopes consists of three accelerations, see (1).

1 $$\begin{aligned} a^v_r = a_e^v + a^v_g - g\sin (\arctan \lambda ) \end{aligned}$$

where $a_e^v$ and $a_g^v$ represent the desired acceleration on a horizontal road and the compensation acceleration on a slope, respectively, with each corresponding to a speed interval of $[v-\Delta v, v+\Delta v]$. The road grade is denoted by $\tan \lambda$. The parameter *g* is the gravitational acceleration.

**Table 1:** Notation.

$a_r^v(k,i)$	The *i*th observed desired acceleration on the *k*th slope in the speed interval
*Δ v*	Half-width of the speed interval $[v-\Delta v, v+\Delta v]$
$a_g^v$	Compensation acceleration in the speed interval *v*
$a_g^j$	The *j*th possible compensation acceleration value
$a_e^v$	Desired acceleration on a horizontal road in the speed interval
*v*	Speed interval $[v-\delta v, v+\delta v]$ centered at speed *v*
*g*	Gravitational deceleration
$\tan \lambda$	Road grade
$\mu _e^v$	Expectation of $a_e^v$
$\sigma _e$	Standard deviation of $a_e^v$
$\mu _g^v$	Expectation of $\ln (a_e^v)$
$\sigma _g^v$	Standard deviation of $\ln (a_e^v)$
$v_f$	Free-flow speed
*β*	Slope of the linear desired acceleration - speed line, on a horizontal road
$\beta _g$	Slope of the linear compensation acceleration - speed line
$\beta _r$	Slope of the linear desired acceleration - speed line on a slope
*k*	Slope number
*θ*	Set of parameter $\theta _k$ across all slopes
	$$\theta =\{\theta _1,\theta _2,...,\theta _k,...\}$$
$\theta _k$	Parameter set $[\beta ,\sigma _e,\mu _g^v(k),\sigma _g^v(k)]$ on slope *k*
$\theta ^*$	Optimized *θ*
$\hat{\theta }$	To be updated *θ* in the EM iterations
$Q^v_i$	Conditional probability $P\left( a^j_{g}|a_r^v(k,i);\theta \right)$
*L(θ )*	Log-likehood with parameter *θ*
*ε*	Convergence threshold
*n*	Number of speed intervals
$\mathrm{{V}}[\cdot ]$	Variance

This decomposition intentionally isolates the gravitational component, which is the dominant and deterministic resistance force on typical highway upgrades. Other resistance forces, such as aerodynamic drag and rolling resistance, are not explicitly separated because they are inherently embedded within the driver’s flat-road desired acceleration model. The model in (1) assumes that the driver’s intrinsic acceleration behavior on a flat road already accounts for the vehicle’s response to these standard resistances. Therefore, the compensation term $a_g$ specifically captures the driver’s additional effort to counteract the novel and substantial force introduced by the slope. This formulation allows us to directly quantify the behavioral adaptation to gradient changes.

Two assumptions are made:

- Assumption 1: The desired acceleration on a horizontal road $a_e$ is stochastic. The expected value of the desired acceleration $a_e$ is denoted by $\mu _e$. The mean desired acceleration would decrease linearly with the speed, see(2).
  2 $$\begin{aligned} \mu _e^v = (v_f-v)\beta \end{aligned}$$
  where the $v_f$ is the free-flow speed. With a given speed *v*, the corresponding desired acceleration $a_e^v$ follows a normal distribution. That is, $a_e^v\sim N(\mu _e^v,\sigma _e)$. $\mu _e^v$ is the expected desired acceleration on a horizontal road section corresponding to speed *v*. This assumption is consistent with the empirical observations in literature^13,17^.
- Assumption 2: with a given speed *v*, the compensation acceleration $a_g^v$ follows a log-normal distribution, i.e, $\ln a_g^v\sim N(\mu _g^v,\sigma _g^v)$. The $\mu _g^v$ and $\sigma _g^v$ are the mean value and standard deviation of $\ln (a_g^v)$. With assumptions, the compensation acceleration would not be negative. Remark here that in the EM-based method we develop here, we can relax this assumption with other distribution function (e.g., normal distribution or Exponential distribution). What specific distribution is the best should be further investigated in the future, but is beyond the goal of this work.

In the problem formulation, the parameter set $\theta _k=(\beta ,\sigma _e,\mu _g^v(k),\sigma _g^v(k))$ cannot not directly measured, hence are called hidden parameters. We classify the acceleration into speed intervals. In one speed interval $[v-\Delta v,v+\Delta v]$, the *i*th observed acceleration is denoted as $a^v_{r}(k,i)$, where *k* means the road slope section number.

### Impacts of compensation acceleration using a linear case study

**Fig. 3:**
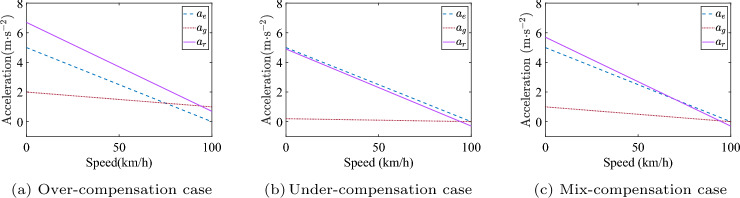
Analyzing impacts of compensation on the accelerations in a linear case. In the numerical study, both $a_e$ and $a_g$ decreases as the speed increases.

In this section, we would like to discuss how the compensatory acceleration affect traffic flow using a linear case study. The linear case means the expected value of $a_g$, i.e., $\mathrm{{E}}(a_g)$, could be described as a linear function of the speed *v*. The aim is to theoretically explore the impacts of compensation accelerations on traffic flow using a special and simple case study.

In the linear case, both expected value of the compensation acceleration $\mathrm{{E}}(a_g)$ and desired acceleration on horizontal road $\mathrm{{E}}(a_e)$ exhibit a direct linear relationship with speed. Consequently, the resultant expected desired acceleration $\mathrm{{E}}(a_r)$ also displays as a linear function of speed. In this section, the compensatory acceleration $\mathrm{{E}}(a_g)$ linearly decreases as the speed increases.

When compensatory forces consistently exceed gravitational losses, the observed desired acceleration $\mathrm{{E}}(a_r)$ curve shifts upward in the acceleration-speed diagram, see Fig. 3(a). Furthermore, under permissible road speed limits, this upward shift may lead to an increase in free-flow speed. It is also possible that the compensation is under-compensation, i.e., the compensation acceleration $\mathrm{{E}}(a_g)<g\cdot \sin (\arctan \lambda )$, the desired acceleration $\mathrm{{E}}(a_r)$ would be shifted downwards in the acceleration-speed diagram (see Fig. 3(b)). In the under-compensation case, the free-flow speed decreases. The mix-compensation case indicates that both sufficient and insufficient compensation are present. In Fig. 3(c), compensation is sufficient at low speeds but becomes insufficient when the speed exceeds 70 km/h. In Fig. 3(c), the free-flow speed reduces and the maximum acceleration increases.

In Fig. 3, the bold line describes the relation between the speed *v* and the observed desired acceleration $a_r$. The slope of the bold line is denoted by $\beta _r$. The variation in $a^v_r$ is denoted as $\mathrm{{V}}(a^v_r)$. As discussed in Laval et al.^17^ and Yuan et al.^20^, two parameters i.e., $\beta _r$ and $\mathrm{{V}}(a^v_r)$, would affect the road capacity drop and oscillation phenomenon. A smaller $\mathrm{{V}}(a^v_r)$ and/or a larger $\beta _r$ would increase the queue discharge rate and stabilize the traffic flow. Since $\beta _r=\beta + \beta _g$, the $\beta _r$ could be increased by adjusting the compensation acceleration. Regarding to the variations, as long as the variation in $\mathrm{{E}}(a_g)$ is positive, the variations in $\mathrm{{E}}(a_r)$ is larger than $\sigma ^2_e$. Consequently, how drivers compensate for gravitational effects on slopes (including the relation between $\mathrm{{E}}(a_g)$ and speed *v*, and the variation of $a^v_g$), significantly impacts road capacity, thereby affecting congestion formation dynamics and clearance efficiency. Therefore, to discuss how a road gradient affect oscillation and capacity is to investigate how the compensatory acceleration and its stochastic nature affect the parameters $\beta _r$ and $\mathrm{{V}}(a^v_r)$.

In our analysis, the driver behavior on slopes is considered as consisting of both a systematic component and a random variation component. Both parts impact slope capacity. If we could control the systematic function and its fluctuations, we might find new ways to manage and optimize capacity on slopes. Building on this, our findings point to an implication for automated systems. If a consistent compensation pattern can be identified using the method developed here, it could directly facilitate the design of autonomous vehicle controls. More specifically, an AV could be programmed to follow a refined, deterministic compensation function. The variations considered as human errors could be controlled or eliminated. More specifically, we might design a compensation acceleration function for automatic vehicles. Consider a linear relation between compensation acceleration $a_g$ and speed *v*, see (3).

3 $$\begin{aligned} \mathrm{{E}}(a_g) = (v_f-v)\beta _g + g\sin (\arctan \lambda ) \end{aligned}$$

Then the acceleration $\mathrm{{E}}(a_r) = (v_f-v)(\beta +\beta _g)$. If both *β* and $\beta _g$ are positive, the $\beta _r$ would increase without affecting the free-flow speed. For automatic vehicles, the human errors (shown as the variations in $a_g$) might be negligible. Then the BM model Laval et al.^17^ could be used to simulate the impacts of slopes. That is, the generated larger $\beta _r$ would facilitate stabilizing traffic flow. Remark here that the real-world effectiveness require future testing with actual AV data.

### Formulations in EM framework

Consider several slopes where data are collected. The slope number is denoted by *k*. In this research, $\{a_r^v(k),v\}$ is a set of known variables on the *k*th slope. The hidden variables are in the set $\theta _k=\left( \beta ,\sigma _e,\mu _g^v(k),\sigma _g^v(k)\right)$ and $\theta =\{\theta _1,\theta _2,...,\theta _k,...\}$. Remark here that *β* and $\sigma _e$ are universal variables for all slopes, since it denotes properties of desired acceleration on horizontal road section. To find the optimal *θ* that maximizes the log-likehood of the observations $\{a_r^v,v\}$, we first formulate the problem in the EM algorithm.

In the following EM formulation, we adopt notation:

- *θ* The complete set of current parameters used within an iteration
- $\hat{\theta }$ The updated set of parameters being solved for in the M-step.
- $\theta ^*$ The final, optimized parameter set after EM convergence.
- The index *j* enumerates candidate values in a discrete set $G =\{a_g^1, a_g^2, ..., a_g^M\}$. This set G approximates the continuous support of the latent variable $a_g$. The summation over j performs a numerical integration over the distribution of $a_g$.

The problem is formulated within two steps:

**E-step:** Given the current parameters *θ*, compute the posterior responsibility for each candidate compensation value $a_g^j$:

4 $$\begin{aligned} Q_i^v(a_g^j; k, \theta ) = P\!\left( a_g^j \mid a_r^v(k,i); \theta \right) \end{aligned}$$

The $Q_i^v(a_g^j; k, \theta )$ are fixed numbers computed for the current iteration. In the subsequent maximization step, they are treated as constants in the optimization over $\hat{\theta }$.

**M-step:** The parameters are updated by maximizing the expected complete-data log-likelihood, where the expectation is taken with respect to the weights computed in the E-step:

5 $$\begin{aligned} \theta ^{*} = \arg \max _{{\theta }} \sum _{k,v,i,j} \underbrace{Q_i^v(a_g^j; k, \theta )}_{\text {Fixed value from (4)}} \cdot \log P\!\left( a_r^v(k,i), a_g^j; {\hat{\theta }}\right) . \end{aligned}$$

In this optimization, the only variables are the parameters in $\hat{\theta }$. The superscript *v* means the acceleration is in a speed interval $[v-\Delta v, v+\Delta v]$. In (5), the $Q^v_{i}(a^j_{g};k,\theta )$ could be considered as the probability of value $\log \frac{P\left( a^v_{r}(k,i),a^j_{g};\hat{\theta }\right) }{Q^v_{i}(a^j_{g};k,\theta )}$. That is, (5) estimates the expected value of $\log \frac{P\left( a^v_{r}(k,i),a^j_{g};\hat{\theta }\right) }{Q^v_{i}(a^j_{g};k,\theta )}$. For each velocity interval, the desired acceleration $a_{r}^v (k,i)$ corresponds to the *i*th observed desired acceleration in the speed interval $[v-\Delta v, v+\Delta v]$ on the *k*th slope. The term $a^j_{g}$ represents the *j*th possible value of $a_g$ from the set *G*, which encompasses all potential values of $a_g$. The set *θ* signifies the solution obtained from the previous iteration, whereas $\hat{\theta }$ denotes the set of parameters to be approximated. The expression $P\left( a^j_{g},a^v_{r}(k,i);\hat{\theta }\right)$ stands for the joint probability distribution of $a^j_{g}$ and $a_{r}^v(k,i)$, which could be expressed as shown in (6).

6 $$\begin{aligned} P\left( a^v_{r}(k,i),a^j_{g};\hat{\theta }\right) = P\left( a^j_{g};\hat{\theta }\right) \cdot P\left( a_r^v(k,i)|a^j_{g};\hat{\theta }\right) \end{aligned}$$

In (5), the probability $Q^v_{i}(a^j_{g};k,\theta )$ is defined as the conditional probability $P\left( a^j_{g}|a_r^v(k,i);\theta \right)$, which can be reformulated as shown in (7).

7 $$\begin{aligned} Q^v_{i}(a^j_{g};k,\theta )=P\left( a^j_{g}|a_r^v(k,i);\theta \right) =\frac{P\left( a^j_{g};\theta \right) \cdot P\left( a_r^v(k,i)|a^j_{g};\theta \right) }{\sum _{j}P\left( a^v_{r}(k,i),a^j_{g};\theta \right) } \end{aligned}$$

where $$\sum _j Q^v_{i}(a^j_{g};k,\theta )=1$$.

According to assumptions in Section “Problem formulation”, the probability of $a_g^j$ and $a_e$ could be formulated by (8) and (9), respectively.

8 $$\begin{aligned} P\left( a^j_{g};\theta \right) =\frac{1}{a_g^j\sqrt{2\pi }\sigma _g^v}e^{-(\ln a_g^j-\mu _g^v)^2/{2{\sigma _g^v}^2}} \end{aligned}$$

and

9 $$\begin{aligned} P\left( a{^v_{e}};\theta \right) =\frac{1}{\sqrt{2\pi }\sigma _e}e^{-(a{^v_e}-\mu _e^v)^2/{2\sigma _e^2}} \end{aligned}$$

where $$\mu _e^v = (v_f-v)\beta$$.

According to (1), we can express $P\left( a_r^v(k,i)|a^j_{g};\hat{\theta }\right) =P(a^v_e=a_r^v(k,i)-a^j_{g}+g\sin (\arctan \lambda );\hat{\theta })$. Hence, combing (1) and (9), the probability $P\left( a_r^v(k,i)|a^j_{g};\hat{\theta }\right)$ could be formulated by (10).

10 $$\begin{aligned} P\left( a_r^v(k,i)|a^j_{g};\hat{\theta }\right) =\frac{1}{\sqrt{2\pi }\hat{\sigma }_e}e^{-\left[ \eta (k,i,v)-(v_f-v)\hat{\beta }\right] ^2/{2\hat{\sigma }_e^2}} \end{aligned}$$

where $$\eta (k,i,v)=a_r^v(k,i)-a^j_{g}+g\sin (\arctan \lambda )$$.

### Solution algorithms

The optimization problem as shown by (5) is solved by using the EM algorithms, which contains two steps: E-step and M-step.

- E-step: Compute $Q^v_{i}(a^j_{g};k,\theta )$ to update the expectation of the complete-data log-likelihood.
- M-step: Maximize the aggregated expectation by solving objective function (5), requiring derivatives of (4) with respect to $\hat{\theta }$. This yields closed-form expressions for the estimated parameters (see (11), (12), (13), and (14)).

11 $$\begin{aligned} \hat{\beta }&= \frac{\sum _k\sum _v\sum _i\sum _jQ_i^v(a_g^j;k,\theta )\cdot \eta (k,i,v)\cdot (v_f-v)}{\sum _k\sum _v\sum _i\sum _jQ_i^v(a_g^j;k,\theta )\cdot (v_f-v)^2} \end{aligned}$$

12 $$\begin{aligned} \hat{\sigma }_e&= \sqrt{\frac{\sum _k\sum _v\sum _i\sum _jQ_i^v(a_g^j;k,\theta )\cdot \left[ (\eta (k,i,v)-(v_f-v)\hat{\beta }\right] ^2}{\sum _k\sum _v\sum _i\sum _jQ_i^v(a_g^j;k,\theta )}} \end{aligned}$$

13 $$\begin{aligned} \hat{\mu }_g^v(k)&= \frac{\sum _v\sum _i\sum _jQ_i^v(a_g^j;k,\theta )\cdot \ln a^j_g}{\sum _v\sum _i\sum _jQ_i^v(a_g^j;k,\theta )} \end{aligned}$$

14 $$\begin{aligned} \hat{\sigma }_g^v(k)&= \sqrt{\frac{\sum _v\sum _i\sum _jQ_i^v(a_g^j;k,\theta )\cdot \left[ \ln a^j_g-\mu _g^v(k)\right] ^2}{\sum _v\sum _i\sum _jQ_i^v(a_g^j;k,\theta )}} \end{aligned}$$

where $\eta (k,i,v)=a_r^v(k,i)-a^j_{g}+g\sin (\arctan \lambda )$. A pseudo code is provided, see Algorithm 1.

**Algorithm 1:**
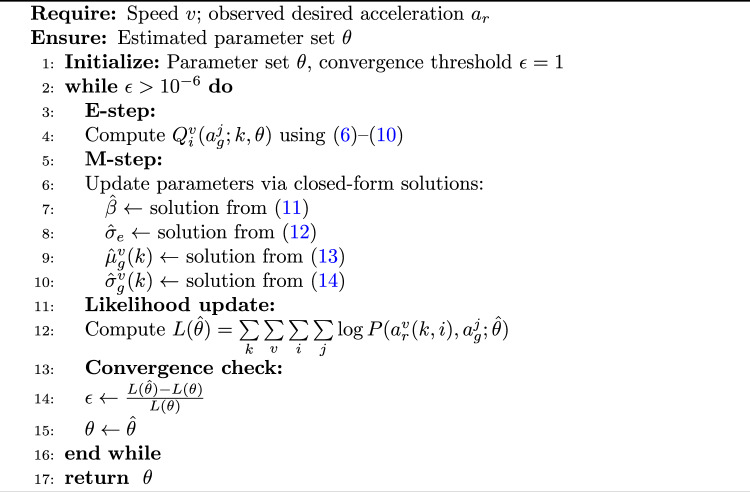
EM Algorithm for Parameter Optimization

## Data and experiments

To demonstrate the proposed method, we applied it on empirical data that are collected in field experiments. The studied data, collected from one driver per vehicle type, are presented as controlled case studies to illustrate the application of the proposed method. They are intended to demonstrate the method’s capability to extract and model compensation behavior under specific conditions, not to characterize the behavior of all drivers of these vehicle types.

In this section, the field experiments are described in Section “Field experiments”. The data are collected and preprocessed in Section "Data and preprocessing".

### Field experiments

**Fig. 4:**
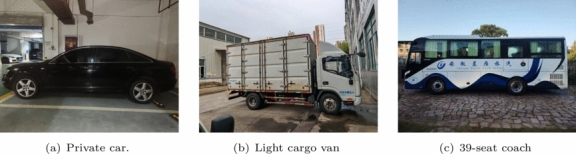
Three types of vehicles are being tested, namely private cars, light cargo van, and coaches.

**Fig. 5:**
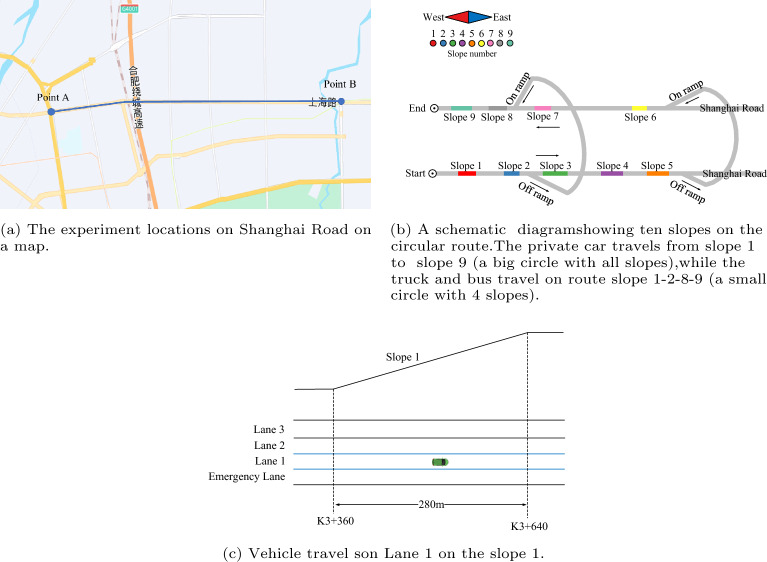
The experiment location on the Shanghai Road Expressway in Hefei City, China. A circular route with ten slopes between the start and the end point.

The experiment aims to collect data on driver-selected acceleration profiles on slopes. High-frequency GPS (0.1-second interval) was utilized to record vehicle trajectories. The test vehicle with an automatic gearbox traveled independently without a preceding leader, maintaining consistent lane position (Lane 1, see Fig. 5((c))) throughout the experiment. All tests were conducted under dry weather conditions during off-peak daylight hours to ensure consistent road geometry, minimize traffic interference, and maintain uniform tire-road friction. At the beginning of each slope, the vehicle decelerated to a low speed (0–5 km/h) before accelerating to the driver’s desired cruising speed. This protocol of initiating acceleration from a near-standstill was intentionally designed to generate a rich acceleration profile across a wide speed range. While it represents a controlled condition rather than a natural slope entry, it provides a comprehensive dataset ideally suited for testing and validating the core capability of the proposed extraction method.The collected data represents single-driver acceleration behavior on free-flow slopes without leading vehicles. Three types of vehicles are being tested, namely private cars, light cargo van, and coaches (see Fig. 4), each with a different driver. The details of tested vehicles and the corresponding drivers are listed in Table 2.

**Table 2:** Information of three types of test vehicles and corresponding drivers.

Vehicle type	Driver	Date	Duration
Private car	Male, 8-year driving experience	December 16–18, 2024; January 22–24, 2025	Six days
Light cargo van, empty	Male, 13-year driving experience	June 9, 2025	Single day
39-seat Coach, empty	Male, 22-year driving experience	June 11, 2025	Single day

**Table 3:** Grade and length of the nine slopes.

Slope number	Grade (%)	Length (m)
1	3.63	280
2	3.95	540
3	3.52	218
4	3.01	365
5	3.83	172
6	3.97	248
7	4.00	282
8	4.00	456
9	3.68	270

The experiments are conducted on Shanghai Road, a major three-lane road in Hefei, China (Fig. 5(a)). The private car travels along a 20 km circular route from location A to B and back to A. The van and coach traverse from slope 1 to slope 2, exit via the off-ramp, and then enter through the on-ramp to go from slope 8 to slope 9. For each type of vehicle, the experimental protocol involved 20 repeated runs by a single male driver, see Table 2. While GPS recorded data continuously, only instances where no vehicles merged in front of the test vehicle were considered valid for analysis. Specifically:

- The route contains nine slopes with specifications (grade and length) detailed in Table 3, verified by the road’s design institute.
- Slope-specific data was only retained when no vehicle merged into the path of the test vehicle.
- The male driver consistently followed the protocol: decelerating to 0–5 km/h at slope start, then accelerating normally through the slope.
- The free-flow speed for the private car, light cargo van and the coach is 100 km/h, 65 km/h and 60 km/h, respectively. The free-flow speed is collected when they are traveling freely on horizontal road sections.

**Fig. 6:**
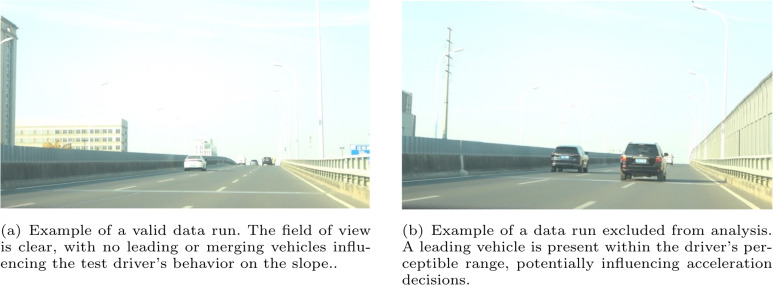
The experiment location on the Shanghai Road Expressway in Hefei City, China. A circular route with ten slopes between the start and the end point.

Before analyzing, we select valid data by screening through post-hoc review of the onboard driving video recordings. A run was excluded if visual inspection of the video confirmed the presence of a leading vehicle within a perceptually influential range during the acceleration phase on the slope, or any interference from merging vehicles. This manual verification ensured that all analyzed trajectories represented free-flow conditions where the driver’s acceleration behavior was a direct response to the slope geometry, not an interaction with other traffic. Fig. 6(a) and (b) exemplify the visual criteria used for inclusion and exclusion, respectively.

The experiment design focuses on intra-driver variability under controlled conditions, examining how the same driver may exhibit different acceleration patterns even when facing identical traffic situations on repeated runs.

Remark here that before conducting the experiment, we fully informed all participants of the potential risks associated with the driving experiment. We clearly instructed each participant to immediately report any discomfort, fatigue, or sense of unsafety, and to stop the experiment at once if such feelings arose. In addition, all subjects provided informed consent before participation.

We would also like to clarify that the experimental process was carried out with careful safety protection. Research staff were actively involved throughout the experiment to ensure operational safety, monitor participant condition, and respond immediately whenever needed.

Before the experiment was implemented, we discussed the safety of the study in details, examined possible risks and hidden hazards, and prepared corresponding preventive measures and emergency plans in advance. Regarding participant eligibility, we have confirmed that all drivers involved in the study possessed valid driving licenses and were legally eligible to drive. Regarding data privacy, all participant data were handled in anonymized form. No personally identifying information was included in the dataset.

**Fig. 7:**
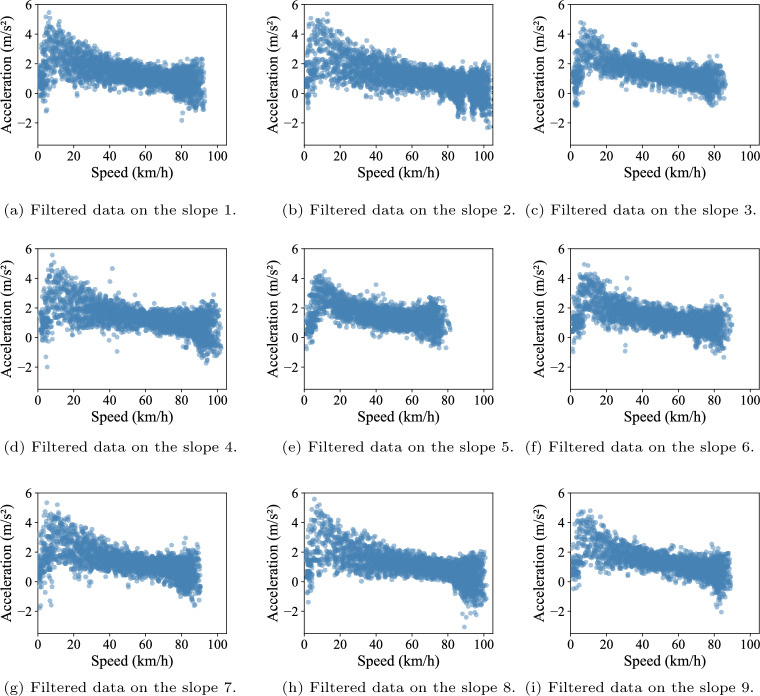
Filtered experiment data. The GPS collects data every 0.1s. The cut-off frequency applied in the Fourier filtering approach in this work is 2Hz.

**Fig. 8:**
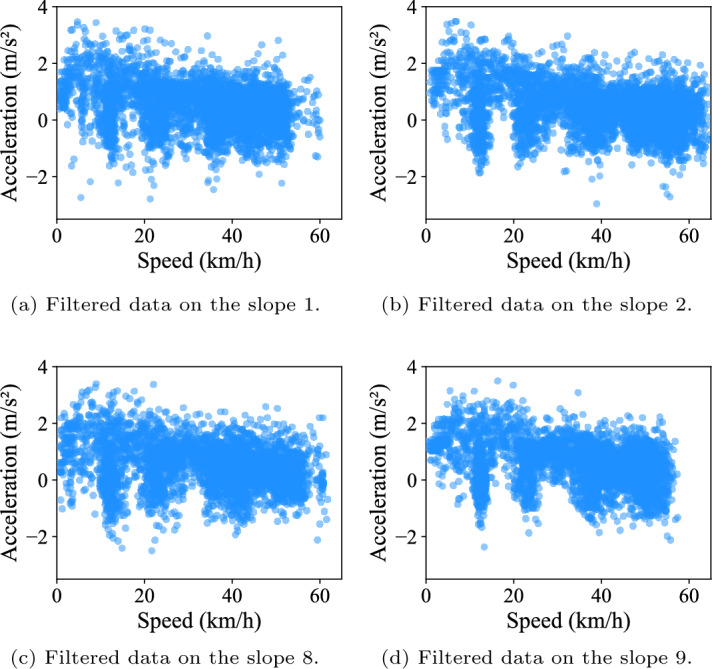
Filtered experiment data for the light cargo van. The GPS collects data every 0.1s. The cut-off frequency applied in the Fourier filtering approach in this work is 2Hz.

**Fig. 9:**
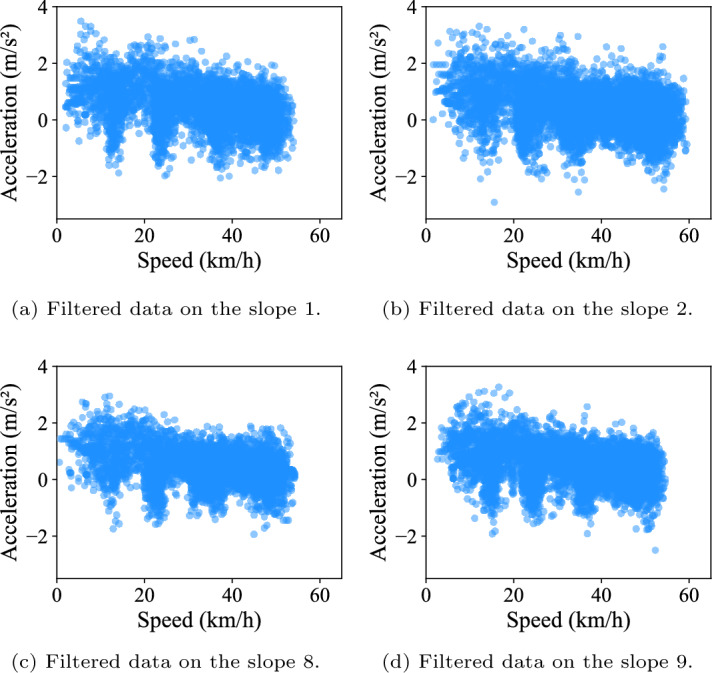
Filtered experiment data for the coach. The GPS collects data every 0.1s. The cut-off frequency applied in the Fourier filtering approach in this work is 2Hz.

### Data and preprocessing

This study focuses particularly on acceleration data. The GPS collects data every 0.1s. The raw GPS data is noisy, leading to unrealistic acceleration values. For example, in the private car experiment, the original $a_r$ ranges between −7$\mathrm {m\cdot s^{-2}}$ and 13$\mathrm {m\cdot s^{-2}}$, with many values exceeding 5$\mathrm {m\cdot s^{-2}}$, which is unrealistic. Literature indicates the maximization acceleration should within [0.5, 5]$\mathrm {m\cdot s^{-2}}$^17,19,20,32,33^. To address this, Fourier filtering^34^ is applied to preprocess the data, filtering speed noise over time to produce more realistic accelerations. The cut-off frequency applied in the Fourier filtering approach in this work is 2Hz. This value is chosen because the frequency band up to 2 Hz contains the majority of information relevant to driver-vehicle longitudinal control dynamics, as established in prior work on trajectory reconstruction for traffic flow theory research^35^. The private car data, light cargo van data and the coach data are presented in Fig. 7, Fig. 8 and Fig. 9, respectively.

As Fig. 7, Fig. 8 and Fig. 9 illustrate, the desired acceleration $a_r$ decreases with increasing speed, aligning with prior findings in^17^. Remark here that the light cargo can data and the coach data would be much more scattered than the private car data. Compared with the private car data, the acceleration of light cargo van and coach would be much lower.

**Fig. 10:**
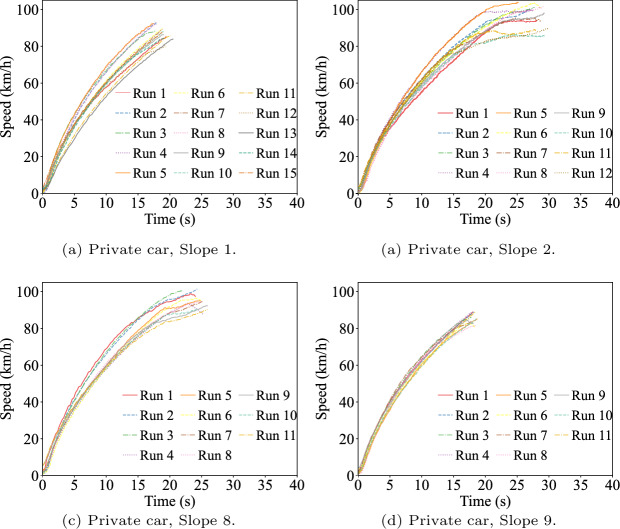
Speed profiles over time of private car on slope 1, 2, 8 and 9. On each slope, they have 15, 12, 14 and 11 valid runs, respectively, due to occasional traffic interference.

For private cars, the speed profiles over time on slope 1, 2, 8 and 9 are given in Fig. 10. On each slope, they have 15, 12, 14 and 11 valid runs, respectively, due to occasional traffic interference. As we can see from Fig. 10, the speed profiles on the same slope exhibit considerable variability. Key parameters such as maximum speed, travel time, and accelerations differ significantly across different runs.

**Fig. 11:**
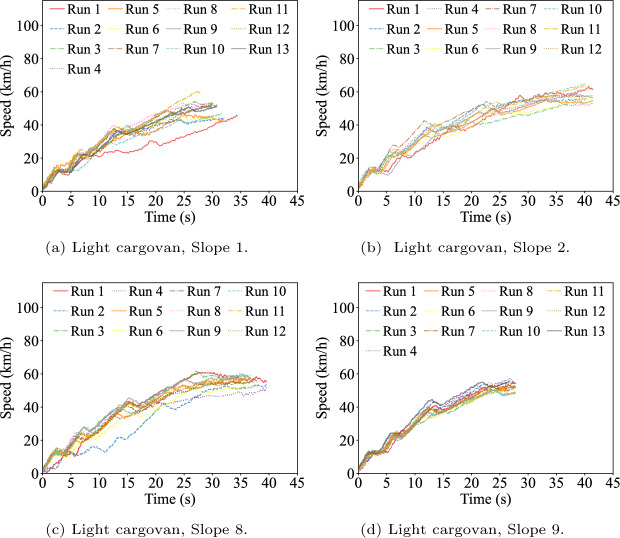
Speed profiles over time of light cargo van on slope 1, 2, 8 and 9. On each slope, they have 13, 12, 12 and 13 valid runs, respectively, due to occasional traffic interference.

**Fig. 12:**
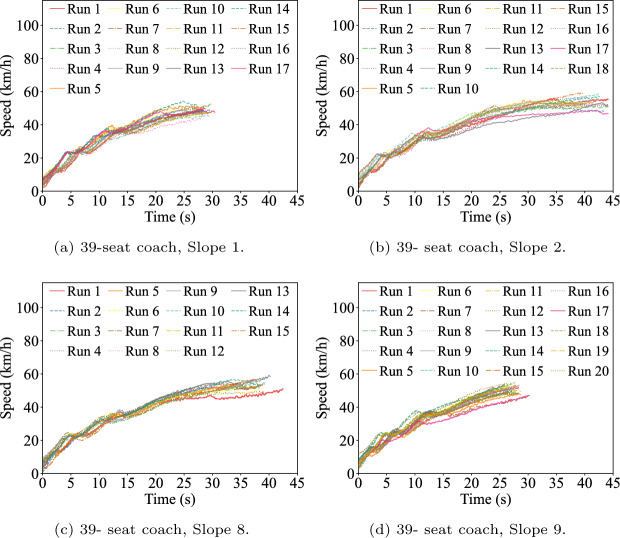
Speed profiles over time of 39-seat coach on slope 1, 2, 8 and 9. On each slope, they have 17, 18, 15 and 20 valid runs, respectively, due to occasional traffic interference.

For the light cargo van and the coach, the speed profiles on four tested slopes are presented in Fig. 11 and Fig. 12, respectively. Similar to the private car data, the light cargo van and coach data demonstrate significant intra-driver variations. In contrast to private cars, the acceleration processes of the light cargo van and the coach tend to be less smooth. Specifically, after speeding up for a period of time, their speeds will decrease. Regarding to possible reasons, for both van and coach, considering the need to stabilize goods and passengers, the acceleration would tend to be less continuous.

**Table 4:** Sample Sizes by Vehicle Type and Slope.

**Slope No.**	**Vehicle Type**	**Number of Data Points**
1	Private car	2,819
	Light cargo van	4,005
	39-seat coach	4,933
2	Private car	3,382
	Light cargo van	4,920
	39-seat coach	7,595
3	Private car	1,925
4	Private car	2,650
5	Private car	2,322
6	Private car	2,520
7	Private car	2,586
8	Private car	2,718
	Light cargo van	3,604
	39-seat coach	4,480
9	Private car	2,008
	Light cargo van	3,590
	39-seat coach	5,592

Table 4 summarizes the number of valid measurements for each vehicle type and slope combination. Private cars cover all nine slopes with sample sizes ranging from 1,925 to 3,382 points. Light cargo vans and 39-seat coaches were tested on four representative slopes (1, 2, 8 and 9), with sample sizes ranging from 3,590 to 4,920, and 4,480 to 7,595, respectively.

## Intra-driver compensation behaviors

We use the empirical experiment data to estimate the intra-driver variation in the compensation acceleration on different slopes. When using the EM algorithms to conduct analysis, the set-up is presented in Section "Set-up". The numerical results are presented in Section “Numerical results”.

### Set-up

When implementing the EM algorithms, several parameters are initialized. The initial value of the inverse relaxation time *β* = 0.15$\mathrm {s^{-1}}$, the initial variance of desired acceleration $\sigma _e$=0.5$\mathrm {m/s^{2}}$, the initial mean of the compensation acceleration $\mu _g^v$ =1.2$\mathrm {m/s^{2}}$, the initial variance of the compensation acceleration $\sigma _g^v$=0.9$\mathrm {m/s^{2}}$. In the iterative process, the convergence threshold *ε* is defined as (15).

15 $$\begin{aligned} \epsilon = [L(\hat{\theta }) - L(\theta )] / L(\theta ) \end{aligned}$$

The iterations will terminate once the tolerance gap satisfies $\epsilon <10^{-6}$. In the EM algorithms, the parameter $a_g$ is selected sequentially from a set *G*. The set *G* is defined as $G = \{1, 11, 21, ..., 100001\}\cdot 10^{-4}$, where values increase by $10^{-3}$ increments. The step size governs the resolution fineness of this numerical approximation. A sensitivity analysis of this parameter is conducted to assess its impact on the computational cost and accuracy.

To validate the estimation results, we generate $10^4$ samples each of $a_e^v$ and $10^4$ samples of $a_g^v$ for every speed interval *v* using estimated parameters and their respective distributions (see Section “Problem formulation”). These simulated accelerations enable calculation of predicted percentiles and expectations. Estimation accuracy is evaluated by comparing predictions against experimental percentiles. The percentage of observation data points in the 80%-percentile band serve as accuracy metric.

To assess the stability and reliability of parameter estimation in the absence of ground truth, we employ a bootstrap resampling procedure. For each driving scenario (slope) and vehicle type, we perform 20 independent bootstrap iterations. In each iteration, we randomly sample 80% of the available measurement sequences without replacement, re-estimate all model parameters via the EM algorithm. This approach allows us to quantify the variability and consistency of our estimates across different subsets of the data.

### Numerical results

In this section, we show numerical results of the compensation acceleration estimations.

#### Model convergence and parameter estimation

**Fig. 13:**
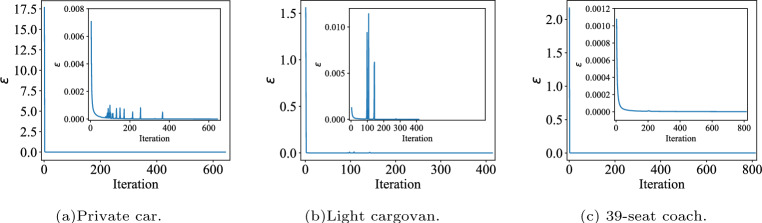
Iterative process of the tolerance gap *ε*. The tolerance gap converges after 661, 414, and 815 iterations for (**a**) the private car, (**b**) light cargo van and (**c**) 39-seat coach, respectively.

The developed EM-based algorithms would finally reach convergence after hundreds of iterations when the accuracy $\epsilon <10^{-6}$, see Fig. 13. The tolerance gap converges after 661, 414, and 815 iterations for the private car, light cargo van and 39-seat coach, respectively.

**Fig. 14:**
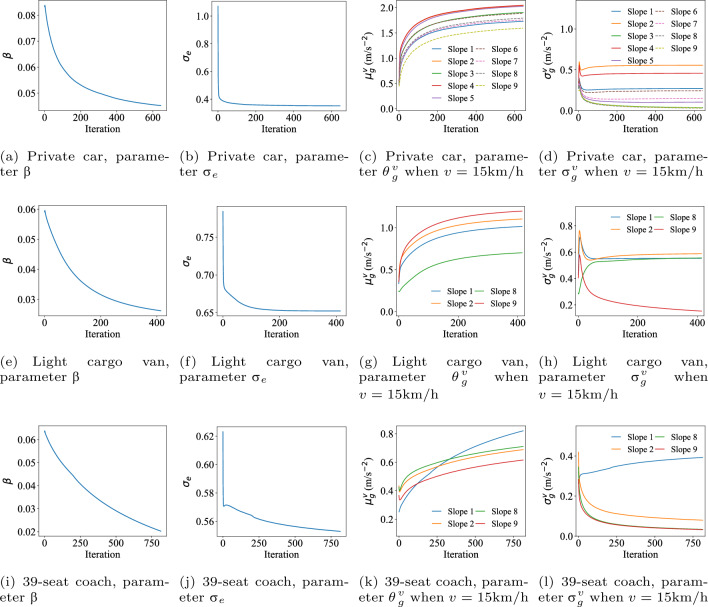
Iterative process for parameters of all three types of vehicles.

**Fig. 15:**
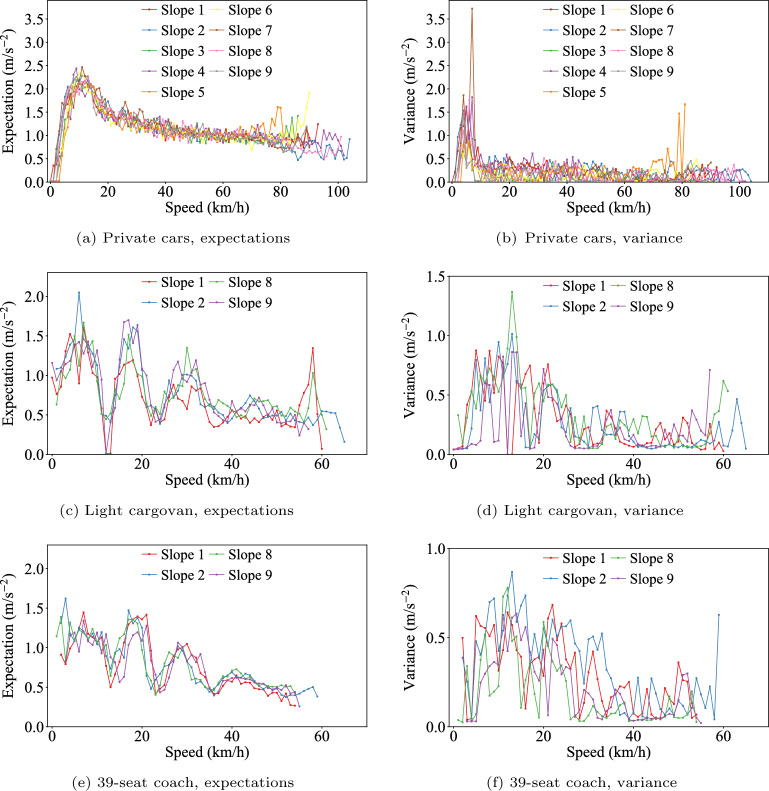
Expectations and variance of compensation acceleration on all tested slopes across all speeds for three types of vehicles.

We show the iteration process for parameter *β*, $\sigma _e$, $\mu _g^v$ and $\sigma _g^v$ at *v=15*km/h for all three types of vehicles in Fig. 14. Each row correspondingly display results for private car, light cargo van and 39-seat coach. As depicted in Fig. 14((a)), ((e)) and ((i)), the iterative gap of *β* across all scenarios reaches approximately $10^{-5}$. Once converged, the parameters *β*, $\sigma _e$ for the three vehicle types settle at (0.045s$^{-1}$, 0.35m/s$^{2}$), (0.026s$^{-1}$, 0.65m/s$^{2}$), and (0.20s$^{-1}$, 0.55m/s$^{2}$) respectively (see Table 5). These parameters reflect the stochastic nature of longitudinal driving behavior on horizontal road sections.

**Table 5:** Estimation results.

Vehicle type	*β* (s$^{-1}$)	$\sigma _e$ (m/s$^{2}$)
Private car	0.045	0.35
Light cargo van	0.026	0.65
39-seat coach	0.020	0.55

We illustrate the convergence of parameters $\mu _g^v$ and $\sigma _g^v$ by showing the convergence process at *v=15* km/h for better readability, see the two columns of Fig. 14 on the far right. Note that both expectation $\textrm{E}(\cdot )$ and variation $\textrm{V}(\cdot )$ in $a^v_g$ could be derived as functions of $\mu ^v_g$ and $\sigma ^v_g$.

When compared to prior experimental findings in the literature, the data for light cargo vans and coaches align with Tu and Laval^13^, and private car data falls within the observation range of Xu and Laval^12^. Unlike Xu and Laval^12^, trucks (including coaches) have a lower *β* value than private cars, indicating less aggressive acceleration by trucks. Consistent with Xu and Laval^12^, trucks exhibit a higher $\sigma _e$ value, implying greater variation in truck drivers’ acceleration. As Xu and Laval^12^ explains, this variation may relate to the number of gears truck engines use during acceleration.

#### Estimated compensation behavior patterns

The stochastic nature of the compensation accelerations are represented by the set of expected value $\mathrm{{E}}(a^v_g)$ and variation $\mathrm{{V}}(a^v_g)$. After the convergence, we present the expected value $\mathrm{{E}}(a^v_g)$ and variation $\mathrm{{V}}(a^v_g)$ of acceleration for three types of vehicles–private cars, light cargo vans, and 39 - seat coaches–across different speeds and slopes, see Fig. 15. Each row correspondingly display results for the private car, light cargo van and 39-seat coach.

- **Private cars.** The expectations generally increase up to a certain speed (around 10 km/h) and then slightly decrease as the speed increases. For variance $\mathrm{{V}}(a^v_g)$, at lower speed the variance is larger than that at higher speed. As the speed exceeds 10 km/h, the variance is below 0.5 m$^2/$s$^4$. This indicates that private car drivers have a relatively wide range of acceleration behaviors at lower speeds. Remark here that the variation $\mathrm{{V}}(a^v_g)$ on slope 5 shows some wide fluctuations when the speed is around 80 km/h. Possibly that is because the number of data points is too small (e.g., only 2 data points in the 80 km/h interval).
- **Light cargo van and 39-seat coach.** For both light cargo vans and 39-seat coaches, the expectation values of acceleration tend to decrease as the speed increases. However, their patterns are less smooth compared to private cars, particularly at some speeds (e.g., around 12 km/h and 22 km/h), the expectation would always be low, regardless of slopes. That may be attributed to differences in driving behaviors or vehicle dynamics. The observed anomalies at 12 km/h and 22 km/h may correspond to potential thresholds in the vehicle’s powertrain strategy on slopes, such as possible gear-holding or downshift points. Remark that this interpretation remain speculative and are derived from a limited sample; they should not be considered definitive conclusions. Instead, they are presented as a preliminary framework to guide future systematic research involving larger, more diverse samples of drivers and vehicles to rigorously investigate uphill driving strategies. Regarding variance, it is relatively high at lower speeds for some slopes, suggesting more variability in acceleration actions. As speed increases, the variance tends to decrease but still shows fluctuations. Numerically, the private car variance would be larger than other two types of vehicles, in terms of both expectations and variance. Arguably, for modeling simplicity and potential applications of our findings, it might be worthwhile to discuss whether a linear function could be used to model the relation between the expected value $\mathrm{{E}}(a^v_g)$ and the speed *v* in the future. It should be noted that the estimated compensation behavior at very low speeds (e.g., <5 km/h) is primarily informed by the induced acceleration regime of our experimental protocol. These estimates should therefore be interpreted as a demonstration of the method’s applicability across speeds within this controlled context, rather than as definitive measures of naturalistic driver behavior at those speeds.

**Fig. 16:**
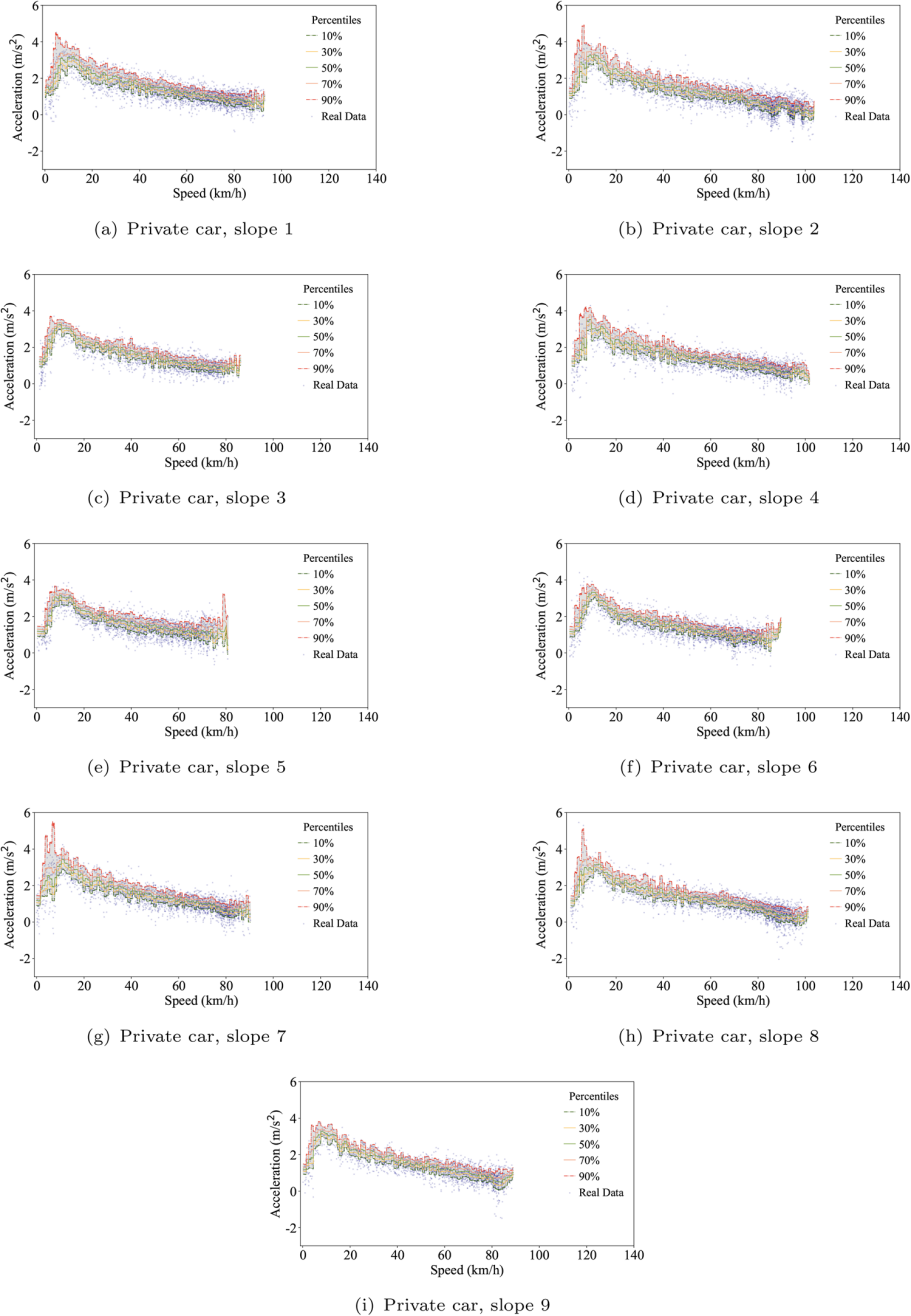
In the private car experiments, comparison between empirical experimental data and the simulated data. The experimental data is presented as dots, while the simulated data are presented as percentiles, that are displayed as lines.

**Fig. 17:**
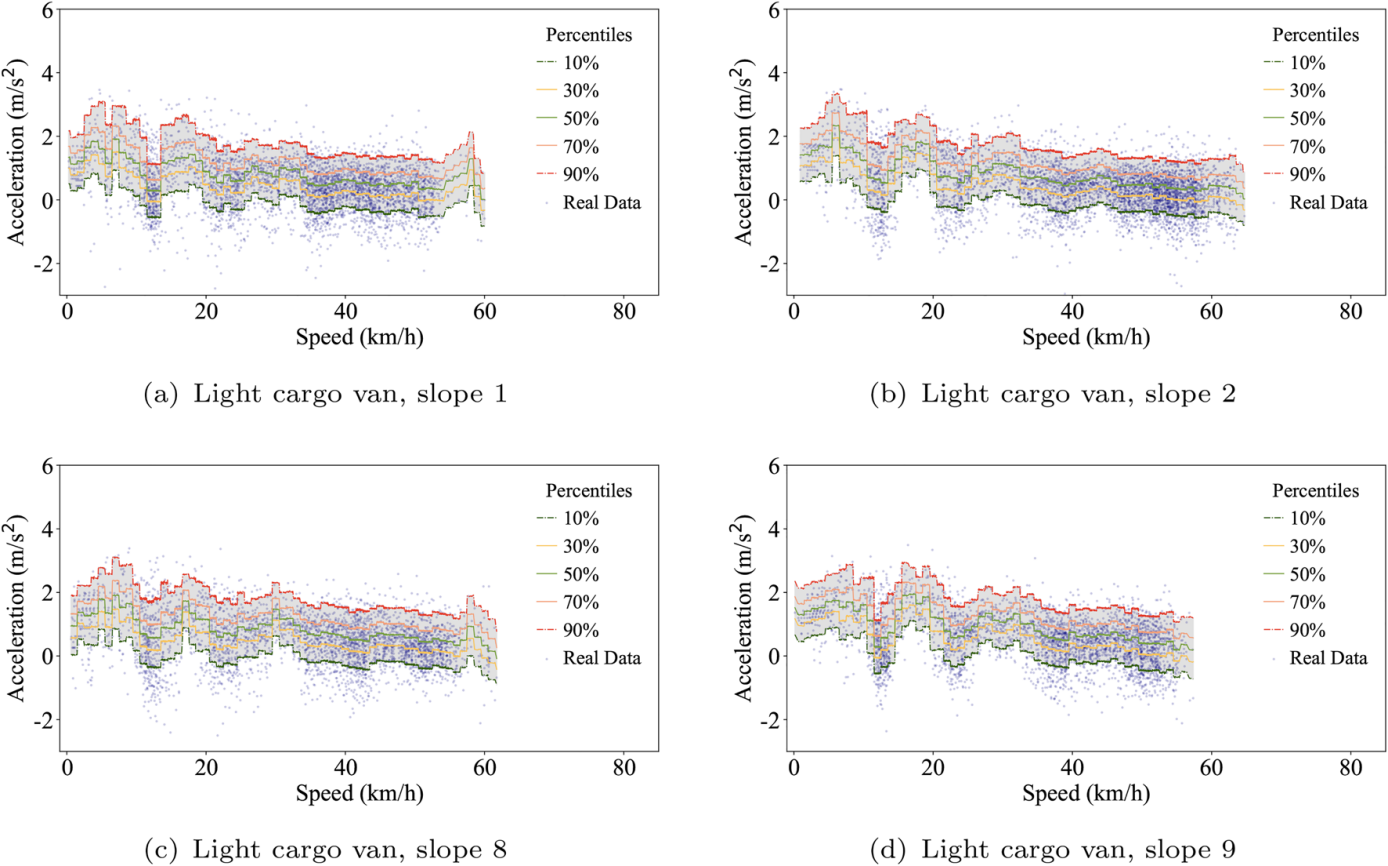
In the light cargo van experiments, comparison between empirical experimental data and the simulated data. The experimental data is presented as dots, while the simulated data are presented as percentiles, that are displayed as lines.

**Fig. 18:**
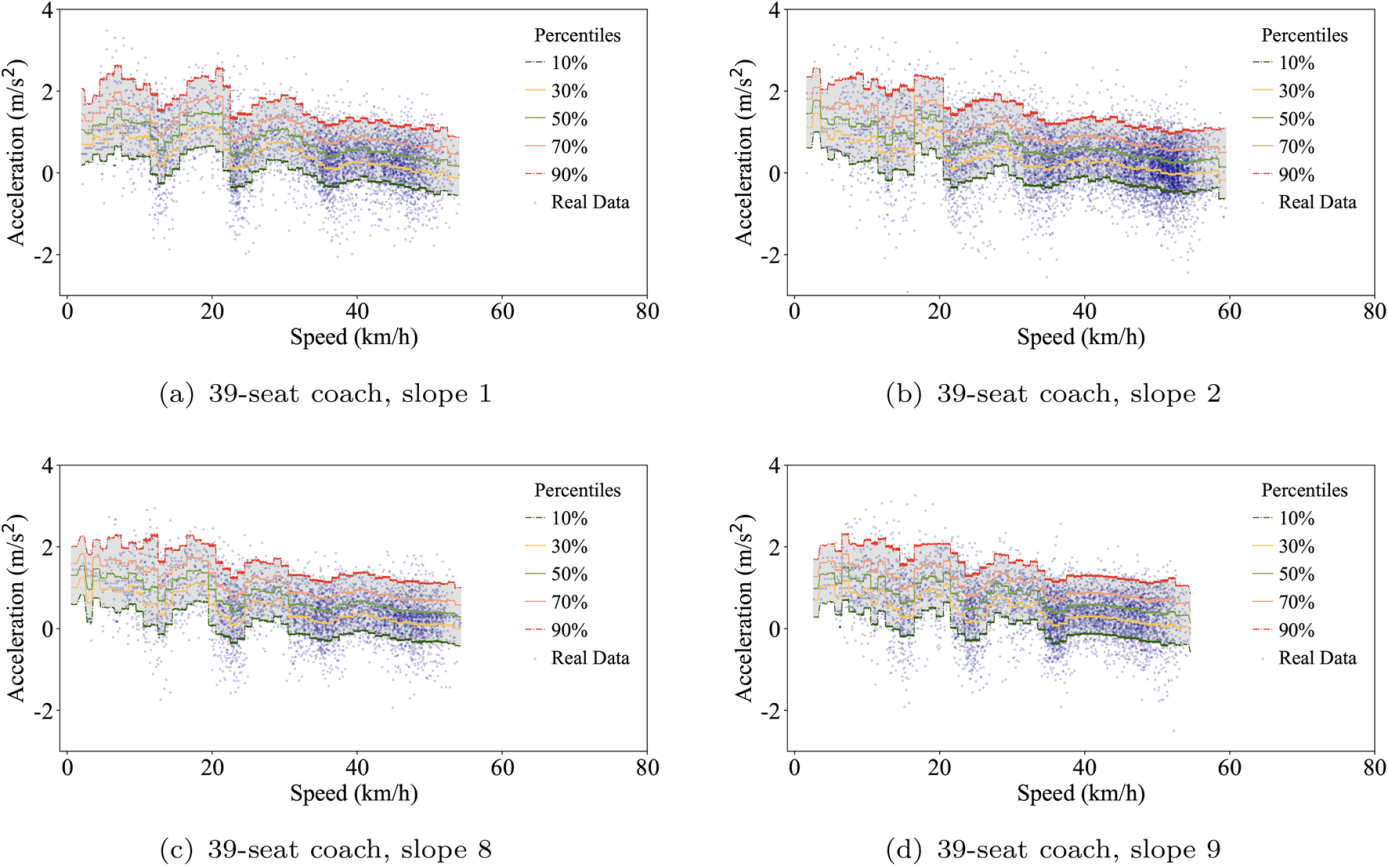
In the 39-seat coach experiments, comparison between empirical experimental data and the simulated data. The experimental data is presented as dots, while the simulated data are presented as percentiles, that are displayed as lines.

#### Model validation

We validate our estimation results using Figure 16 through Figure 18, which display results for the private car, light cargo van, and coach, respectively. Experimental data are shown as dots in these figures, while predicted percentiles (10th, 30th, 50th, 70th, and 90th) are plotted using estimated parameters. The shaded area between the 10th and 90th percentiles represents an 80%-percentile band.

**Fig. 19:**
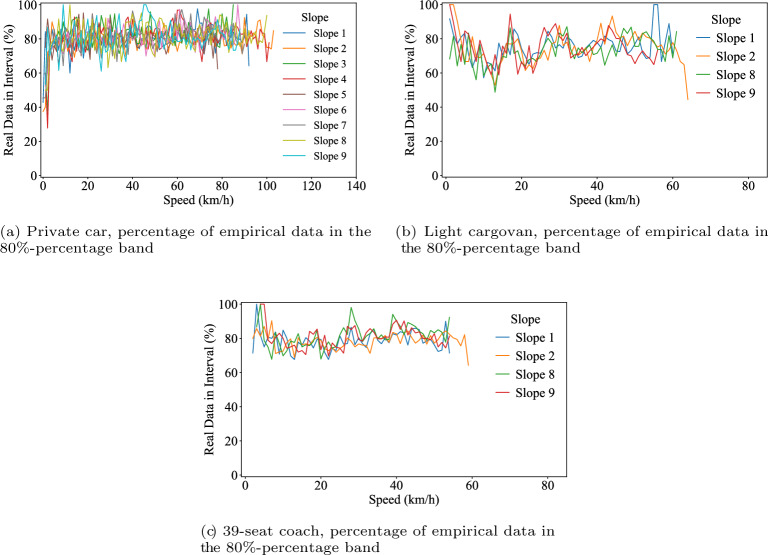
Comparisons between empirical data and the predicted 80%-percentage band.

Most experimental data points fall within the predicted 80%-percentile band. To quantify this agreement, we calculate the percentage of data points within the band (Figure 19). Results show that approximately 80% of empirical data reside within the band on average across vehicle types, with detailed distributions for private cars, light cargo vans, and 39-seat coaches shown in Figure 19((a)), 19((b)), and 19((c)), respectively. While the percentage remains near 80% across most speed ranges, private cars exhibit reduced coverage (40%) below 5 km/h. As shown in Figure 16, most deviant points fall below the band during these low-speed intervals.

This phenomenon can be attributed to the relationship between the actual acceleration ($a_r$) and the predicted acceleration components. Specifically, $a_r=a_e+a_g-g\sin \arctan \lambda$. In cases where $a_g$ approaches zero (e.g., at near-zero speeds), the resulting 80%-percentile band is still higher than the actual data. This suggests that the predicted $a_e$ is larger than the actual acceleration. This discrepancy is likely related to Assumption 1, which assumes that the desired acceleration would be the highest at near-zero speeds. However, in reality, vehicle dynamics might constrain the desired acceleration magnitudes, leading to a deviation between the predicted and actual acceleration values.

**Fig. 20:**
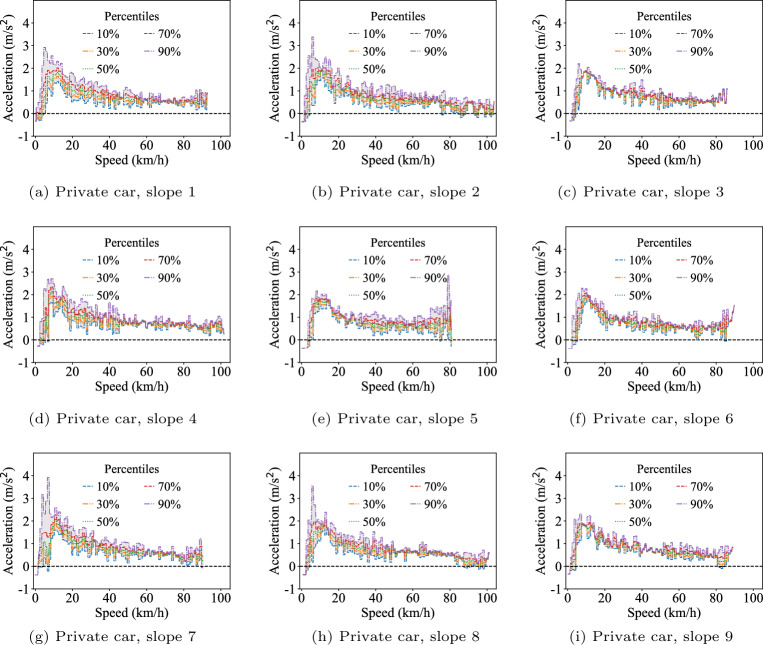
Measured excess acceleration $a_g - g\sin \left( \arctan \lambda \right)$ versus speed for the private car experiments.

**Fig. 21:**
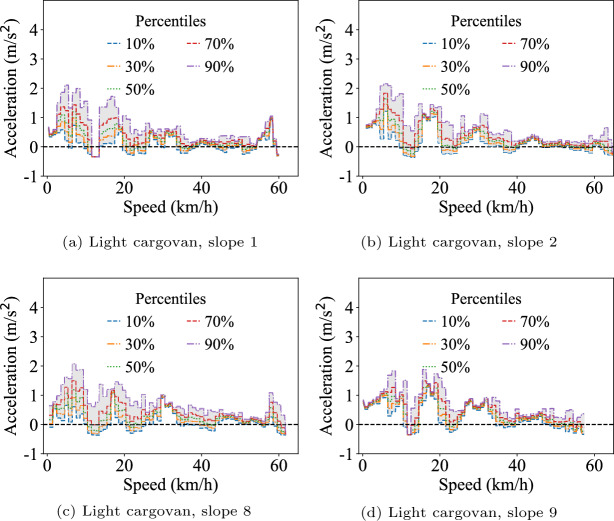
Measured excess acceleration $a_g - g\sin \left( \arctan \lambda \right)$ versus speed for the light cargo van experiments.

**Fig. 22:**
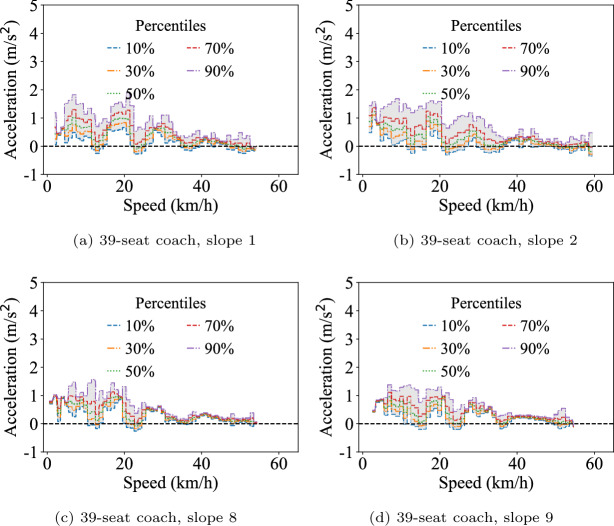
Measured excess acceleration $a_g - g\sin \left( \arctan \lambda \right)$ versus speed for the 39-seat coach experiments.

#### Analysis of compensation sufficiency

Using the estimated parameters, we investigate whether compensation acceleration sufficiently offsets gravitational loss. Figures 20 to 22 display the difference $a_g^v-g\sin \left( \arctan \lambda \right)$ for all three vehicle types, where $a_g^v$ is generated from estimated parameters following the assumed distribution. At each speed interval *v*, $10^4$ samples of $a_g^v$ are simulated. The 10th, 30th, 50th, 70th, and 90th percentiles are shown as lines, with the shaded area between the 10th and 90th percentiles representing an 80%-percentile band. When this band lies entirely above 0 m/s$^2$, it indicates *⩾*90% probability of over-compensation.

For private cars (Fig. 20), the 80%-percentile band consistently exceeds zero, indicating predominant over-compensation. This driving style may explain high acceleration values $a_r$ that are higher than 3 m*·*s$^{-2}$, (see Fig. 7). We note these private car results may be driver-specific, warranting future investigation of driver heterogeneity.

In contrast, light cargo vans and coaches (Figs. 21, 22) exhibit dynamic compensation sufficiency. Significant insufficiency occurs at 15 km/h and 22 km/h, where the 50th-percentile falls below 0 m/s² (indicating *⩾*50% probability of insufficiency). Above 50 km/h, differences converge to zero with reduced variance. Remark here that fluctuations is substantially stronger than in private cars.

Comparatively, slope effects manifest differently across vehicle types: Private cars exhibit systematic over-compensation, while vans and coaches demonstrate fluctuating, discontinuous compensation patterns that may exacerbate instability and capacity reductions. Notably, despite differing vehicle types and drivers, light cargo vans and 39-seat coaches show remarkably similar compensation behaviors. This similarity suggests that investigations of driving style heterogeneity should prioritize private cars.

#### Bootstrapping and sensitivity analysis

Table 6 presents the bootstrap consistency analysis for all estimated parameters across vehicle types and gradient ranges. For each combination, we report the mean, variance, and coefficient of variation (CV) computed over 20 bootstrap iterations. The CV, defined as the ratio of the standard deviation to the mean, serves as our primary measure of estimation stability: lower CV values indicate higher consistency across resampled datasets.

For the private car, the global parameters *β* and $\sigma _e$ exhibit high stability with CVs of 1.53% and 0.62% respectively. For slope-specific parameters, $\mu _g$ shows moderate variability (CVs ranging from 2.85% to 6.88%), while $\sigma _g$ maintains consistent stability across all slopes (CV = 6.93%). For the 39-seat coach, the global parameters show slightly higher variability (*β* CV = 5.23%, $\sigma _e$ CV = 4.78%). The $\mu _g$ parameter demonstrates the highest variability among vehicle types (CVs from 7.80% to 7.91%), reflecting greater estimation uncertainty in coach scenarios. For the light cargo van, with CVs of 4.87% for *β*and 4.12% for $\sigma _e$, the light cargo van shows intermediate stability. Notably, $\mu _g$ maintains consistent variability across all slopes (CV = 6.83%). The coefficients of variation below 8% for all parameters indicate acceptable estimation consistency given the inherent uncertainties in applications. The observed stability patterns align with vehicle-specific operational characteristics, i.e., the test private car demonstrates the highest stability, followed by the test light cargo van and the 39-seat coach.

**Table 6:** Bootstrap Statistics of Estimated Parameters over 20 Iterations.

Vehicle type	Slope No.	**Mean**				**Variance**				**CV (%)**			
		*β*	$\sigma _e$	$\mu _g$	$\sigma _g$	*β*	$\sigma _e$	$\mu _g$	$\sigma _g$	*β*	$\sigma _e$	$\mu _g$	$\sigma _g$
Car	1	0.05	0.38	1.41	0.21	$6.11{\times }10^{-7}$	$5.49{\times }10^{-6}$	$5.90{\times }10^{-3}$	$2.18{\times }10^{-4}$	1.53	0.62	5.45	6.93
	2			1.74	0.50			$8.05{\times }10^{-3}$	$1.21{\times }10^{-3}$			5.15	6.93
	3			1.61	0.04			$2.22{\times }10^{-3}$	$6.40{\times }10^{-6}$			2.94	6.93
	4			1.73	0.42			$1.41{\times }10^{-2}$	$8.51{\times }10^{-4}$			6.88	6.93
	5			1.68	0.10			$2.30{\times }10^{-3}$	$5.26{\times }10^{-5}$			2.85	6.93
	6			1.60	0.18			$5.44{\times }10^{-3}$	$1.48{\times }10^{-4}$			4.62	6.93
	7			1.47	0.10			$3.19{\times }10^{-3}$	$4.59{\times }10^{-5}$			3.84	6.93
	8			1.51	0.03			$3.77{\times }10^{-3}$	$4.80{\times }10^{-6}$			4.07	6.93
	9			1.28	0.03			$5.43{\times }10^{-3}$	$5.82{\times }10^{-6}$			5.76	6.93
Coach	1	0.02	0.55	0.78	0.38	$1.26{\times }10^{-6}$	$6.99{\times }10^{-4}$	$3.80{\times }10^{-3}$	$8.31{\times }10^{-4}$	5.23	4.78	7.91	7.64
	2			0.66	0.09			$2.68{\times }10^{-3}$	$4.33{\times }10^{-5}$			7.80	7.64
	8			0.69	0.04			$3.01{\times }10^{-3}$	$6.29{\times }10^{-6}$			7.91	6.96
	9			0.61	0.03			$2.29{\times }10^{-3}$	$7.11{\times }10^{-6}$			7.90	7.64
Cargo	1	0.03	0.65	1.03	0.58	$1.49{\times }10^{-6}$	$7.15{\times }10^{-4}$	$4.91{\times }10^{-3}$	$1.71{\times }10^{-3}$	4.87	4.12	6.83	7.16
	2			1.11	0.57			$5.76{\times }10^{-3}$	$1.67{\times }10^{-3}$			6.83	7.16
	8			0.73	0.56			$2.51{\times }10^{-3}$	$1.63{\times }10^{-3}$			6.83	7.16
	9			1.19	0.16			$6.65{\times }10^{-3}$	$1.27{\times }10^{-4}$			6.83	7.16

Note: CV = Coefficient of Variance. Parameters*β*and$\sigma _e$are global (shared across all slopes within each vehicle type). The values in merged cells are identical across slopes.

**Fig. 23:**
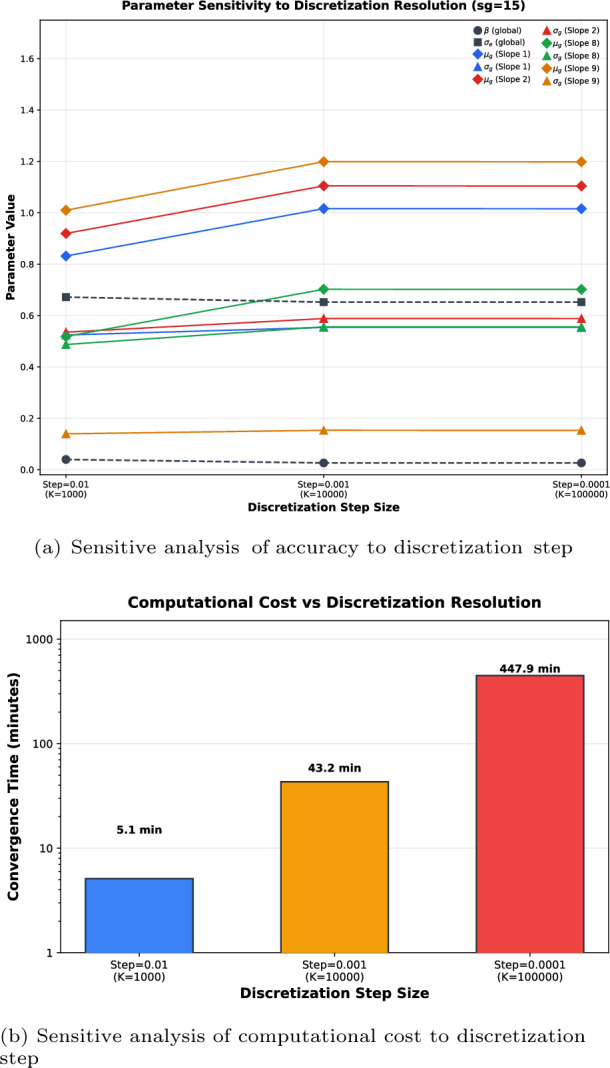
Sensitive analysis to the discretization scheme.

A sensitivity analysis confirmed that the chosen discretization step size (0.001 m/s$^2$) represents an optimal trade-off. The estimated parameters stabilize at this resolution, with changes to a finer step (*Δ* = 0.0001 m/s$^2$) yielding negligible differences ($\leqslant 1\%$) (see Fig. 23(a))while incurring a tenfold increase in computational cost (see Fig. 23(b)). A coarser step (*Δ* = 0.01 m/s$^2$) introduces noticeable bias (up to 15%). Therefore, the main findings are robust to discretization given the sufficient resolution employed.

## Conclusions and future research

This paper presents a comprehensive method for estimating compensation acceleration across different speeds on slopes, focusing on three distinct vehicle types: private cars, light cargo vans, and coaches. Our approach, grounded in the Expectation-Maximization (EM) algorithm, harmoniously blends rule-based models with data-driven learning to capture the stochastic nature of driving behaviors on slopes. The rule-based model delineates the relationship between desired acceleration and speed, while the EM algorithm is employed to infer the parameters of these models from real-world data, a methodology also recognized as a gray-box model in Jung et al.^31^.

Compared with prior work Xu and Laval^17^; Tu and Laval^12^; Laval et al.^13^, this study’s novelty is methodological and conceptual. Laval et al.^17^ provided the stochastic foundation for flat-road driving, and Xu and Laval^12^; Tu and Laval^13^ quantified the compensation effect into a single static parameter. The key advancement in this study is the shift from treating compensation as a fixed parameter to modelling it as a speed-dependent stochastic variable. By estimating both its expectation and variance, we provide a tool, that enables future research to investigate how the dynamics of driver behaviour influence macro-scale traffic phenomena, such as the severity and formation of bottlenecks on graded highways.

### Findings

This analysis of our experimental data for the three vehicle types indicates that both the expected value and variation of compensation acceleration broadly decline as speed increases. While this general trend is observable across private cars, light cargo vans, and coaches, specific patterns exhibit noticeable variations, likely attributable to differences in vehicle dynamics and individual driving behaviors. One potential avenue for future work would be to explore whether a linear function can suitably model the relationship between the expected compensation acceleration ($a_g^v$) and speed. A positive slope ($\beta _g$) in such a model could be theorized to support traffic flow stability and improve queue discharge. Conversely, a significant variance ($V(a_g^v)$) might contribute to greater variability in the overall desired acceleration ($V(a_r^v)$), which could introduce instability and reduce discharge efficiency in dense traffic. The net effect of compensation behavior on slope capacity appears to depend on a combination of driving tendency (over- or under-compensation) and the inherent stochasticity of the behavior, suggesting a complex interaction that merits further investigation.

The proposed method demonstrates promising reliability. For all vehicle types, approximately 80% of the empirical data points fall within the 80% percentile band (between the 10th and 90th percentiles). The EM algorithm converged robustly ($\epsilon < 10^{-6}$) across cases, yielding parameter estimates that align with established empirical benchmarks: private cars ($\beta = 0.045 \, \text {s}^{-1}$, $\sigma _e = 0.35 \, \text {m/s}^2$), light cargo vans ($\beta = 0.026 \, \text {s}^{-1}$, $\sigma _e = 0.65 \, \text {m/s}^2$), and 39-seat coaches ($\beta = 0.020 \, \text {s}^{-1}$, $\sigma _e = 0.55 \, \text {m/s}^2$). The larger $\sigma _e$ values for vans and coaches reflect their higher observed acceleration variability, a finding consistent with prior literature.

The specific results from our limited dataset suggest behavioral differences among vehicle types. The acceleration profiles for private cars, for instance, indicate a tendency toward over-compensation across the tested slopes, with the 80% percentile band for $a_g - g\sin (\arctan \lambda )$ predominantly above zero. In contrast, light cargo vans and coaches showed more dynamic compensation, with instances of under-compensation at specific speeds (e.g., near 15 km/h and 22 km/h), where the median value fell below zero. Their acceleration patterns also appeared less smooth compared to private cars, with variance particularly notable at lower speeds on certain slopes. These patterns may arise from distinct vehicle dynamics or driving styles associated with commercial vehicle operation.

### Discussions

Since ground-truth values are unavailable, the bootstrap analysis reported in Table 6 provides a principled means of evaluating model reliability. The observed coefficients of variation (CV) for all parameters remain below 8% across most vehicle-slope combinations, indicating consistent parameter estimation under repeated 80% data resampling. This consistency supports the robustness of the proposed stochastic compensation framework. While bootstrap stability cannot directly substitute for supervised metrics such as RMSE, demonstrating low estimation variability represents a widely accepted validation practice in scenarios where the target quantities are inherently unobservable.

Table 4 reveals considerable variation in sample sizes across vehicle types and slopes. Although such imbalance may raise concerns about estimation bias, our bootstrap analysis confirms that parameter estimates remain consistent regardless of sample size. This indicates that the current data are adequate for stable estimation and that the impact of uneven data distribution is practically negligible.

In this work, our numerical analysis reveals several empirical characteristics of compensation behavior. These findings contribute to a deeper understanding of this phenomenon. For instance, the observed tendency for over-compensation could offer a plausible explanation for why certain slopes do not function as traffic bottlenecks. However, it is critical to acknowledge the primary limitation of this study, i.e., the data are derived from only one driver per vehicle type. This experimental design was suitable for a proof-of-concept phase, serving to validate the method under controlled conditions. Consequently, the specific numerical results presented should be interpreted as illustrative of the method’s application and the behavior of these specific driver-vehicle units. It is not suggested to consider the numerical findings as generalizable results representing all drivers of these vehicle classes. The core contribution remains the methodological framework, which establishes the foundation for future large-scale studies involving multiple drivers to derive statistically representative findings.

A key limitation of the current study is its focus on free-flow driving behavior. The model does not capture how compensation behavior interacts with vehicle-to-vehicle responses in congested traffic, which is critical for bottleneck analysis. It would be interesting to validate a hypothesis that an over-compensation with low variation could improve queue discharge rates, but the aggressive driving style might correlate with stop-and-go waves.

In this work, two assumptions are made. For the first assumption, it is necessary to remark that the accuracy of (2) may decrease during transient phases such as initial acceleration from very low speeds (e.g., *< 5* km/h). The framework developed herein may be extended to more complex formulations (e.g., piecewise linear) for applications requiring full-range modeling. For assumption 2, the log-normal distribution ensures the non-negativity of compensation acceleration and, permits closed-form parameter updates within the EM algorithm. While other non-negative distributions (e.g., truncated normal) are conceptually valid, numerical optimization might also be needed in each M-step. That would add computational complexity. A systematic comparison of distributional fits remains a valuable direction for future research to determine the optimal statistical characterization of the stochastic compensation behavior.

A potential concern in repeated-run experiments is the influence of learning effects, whereby drivers may become increasingly familiar with the slope geometry and adjust their compensation behavior accordingly. In this proof-of-concept study, such effects-if present-do not undermine the methodological contribution. The proposed EM framework consistently recovered interpretable speed-dependent compensation patterns, as evidenced by stable convergence and bootstrap consistency. Moreover, the experimental design mitigated strong learning effects through multi-day scheduling, moderate run counts, and adequate rest intervals. Future work using naturalistic driving data (where drivers encounter slopes without repeated presentation of slopes) will be better positioned to quantify learning effects explicitly. Nevertheless, the current results could be considered as an early step to study the compensation behavioral on slopes.

### Future directions

In the future, a logical next step is to apply this method to a large-scale naturalistic driving dataset to quantitatively assess how the stochastic properties of driver compensation behavior collectively influence emergent phenomena. That is, upgrade bottleneck capacity, discharge rates, and the formation and propagation of stop-and-go waves.

As suggested by our findings, the compensation behavior is the outcome of an interplay among the driver, the vehicle, and the road. In our current model, vehicle dynamics are exogenous. A future extension would be to tag trajectory data with vehicle attributes (e.g., power-to-weight ratio) and road design parameters, and to explicitly model the compensation parameters as functions of these physical variables. This would allow us to quantify how the stochastic compensation behavior co-varies with both vehicle type and road geometry. For instance, it could allow engineers to estimate the capacity impact of a new vehicle type on an existing slope, or to optimize a new road design for a projected future vehicle fleet.

Given the limited data set from field experiments, future research should focus on collecting more comprehensive data related to compensatory behaviors on slopes. Drones, which have been used to collect trajectory data, offer a potential solution. Different from our field experiments, the data captured by drones is from traffic conditions on roads. The acceleration is experienced accelerations, which would be affected by leading vehicles and congestion in the downstream. This approach has an advantage in that the experienced accelerations would provide much larger sample sizes. However, it also presents a challenge: how to accurately derive the intra-driver desired compensatory acceleration from the experienced acceleration. There are two main difficulties. First, the experienced acceleration can be influenced by the leading vehicle. Second, it is challenging to track the same driver across different days or runs. Besides, in the future, the authors are working on investigating the interplay between the compensatory acceleration behavior and the slope feature (i.e., grade and length). Finally, whether and how the speed limit impacts the interplay is also to be investigated in the future.

We acknowledge that repeated exposure to the same slope segments may still influence driver behavior through learning or habituation, which represents a limitation of the current experimental design. Future studies employing naturalistic driving data or controlled experiments with a larger pool of drivers and slopes could help assess the potential influence of learning effects on estimated compensation parameters.

## Data Availability

Data available on request from the authors.
